# AdpA, a developmental regulator, promotes ε-poly-l-lysine biosynthesis in *Streptomycesalbulus*

**DOI:** 10.1186/s12934-022-01785-6

**Published:** 2022-04-09

**Authors:** Rui Huang, Honglu Liu, Wanwan Zhao, Siqi Wang, Shufang Wang, Jun Cai, Chao Yang

**Affiliations:** 1 Key Laboratory of Molecular Microbiology and Technology, Ministry of Education, College of Life Sciences, Nankai University, Tianjin, 300071 China; 2 Key Laboratory of Bioactive Materials, Ministry of Education, College of Life Sciences, Nankai University, Tianjin, 300071 China

**Keywords:** AdpA, *Streptomycesalbulus*, ε-Poly-l-lysine, Polymerization degree, Morphological differentiation

## Abstract

**Background:**

AdpA is a global regulator of morphological differentiation and secondary metabolism in *Streptomyces,* but the regulatory roles of the *Streptomyces* AdpA family on the biosynthesis of the natural product ε-poly-l-lysine (ε-PL) remain unidentified, and few studies have focused on increasing the production of ε-PL by manipulating transcription factors in *Streptomyces*.

**Results:**

In this study, we revealed the regulatory roles of different AdpA homologs in ε-PL biosynthesis and morphological differentiation and effectively promoted ε-PL production and sporulation in *Streptomyces* *albulus* NK660 by heterologously expressing *adpA* from *S.* *neyagawaensis* NRRLB-3092 (*adpA*_*Sn*_). First, we identified a novel AdpA homolog named AdpA_*Sa*_ in *S.* *albulus* NK660 and characterized its function as an activator of ε-PL biosynthesis and morphological differentiation. Subsequently, four heterologous AdpA homologs were selected to investigate their phylogenetic relationships and regulatory roles in *S.* *albulus*, and AdpA_*Sn*_ was demonstrated to have the strongest ability to promote both ε-PL production and sporulation among these five AdpA proteins. The ε-PL yield of *S.* *albulus* heterologously expressing *adpA*_*Sn*_ was approximately 3.6-fold higher than that of the control strain. Finally, we clarified the mechanism of AdpA_*Sn*_ in enhancing ε-PL biosynthesis and its effect on ε-PL polymerization degree using real-time quantitative PCR, microscale thermophoresis and MALDI-TOF–MS. AdpA_*Sn*_ was purified, and its seven direct targets, *zwf*, *tal*, *pyk2*, *pta*, *ack*, *pepc* and a transketolase gene (DC74_2409), were identified, suggesting that AdpA_*Sn*_ may cause the redistribution of metabolic flux in central metabolism pathways, which subsequently provides more carbon skeletons and ATP for ε-PL biosynthesis in *S.* *albulus*.

**Conclusions:**

Here, we characterized the positive regulatory roles of *Streptomyces* AdpA homologs in ε-PL biosynthesis and their effects on morphological differentiation and reported for the first time that AdpA_*Sn*_ promotes ε-PL biosynthesis by affecting the transcription of its target genes in central metabolism pathways. These findings supply valuable insights into the regulatory roles of the *Streptomyces* AdpA family on ε-PL biosynthesis and morphological differentiation and suggest that AdpA_*Sn*_ may be an effective global regulator for enhanced production of ε-PL and other valuable secondary metabolites in *Streptomyces*.

**Supplementary Information:**

The online version contains supplementary material available at 10.1186/s12934-022-01785-6.

## Background

Two notable characteristics of *Streptomyces* species are the ability to produce multitudinous valuable secondary metabolites possessing diverse biological activities and a complex life cycle, including the generation of vegetative mycelium, aerial mycelium and spores during development [1–3]. The processes of secondary metabolism and morphological differentiation are tightly controlled by multiple levels of transcriptional regulators that include cluster-situated, pleiotropic, and global regulators in response to numerous physiological and environmental conditions [3–6].

The AraC family transcription factor known as AdpA is universally present in *Streptomyces*, is able to amplify the A-factor signal and is a global regulator of morphological differentiation and secondary metabolism in *Streptomyces* [7–10]. AdpA contains a ThiJ/PfpI/DJ-1-like (GATase-1) domain for dimerization and an AraC/XylS family DNA-binding domain, located at its N- and C-termini, respectively, and has an average length of 320–345 aa in *Actinobacteria* [7, 11]. It was demonstrated that the AdpA regulon encompassed 100 to 500 genes in different *Streptomyces* species [10, 12, 13]. AdpA plays a central role in the regulation of morphological differentiation in most *Streptomyces* species [3, 14–18]. On the one hand, AdpA positively regulates the transcription of direct targets involved in morphological differentiation in *Streptomyces* *griseus*, such as *ssgA* encoding a protein that forcefully influences septum formation, σ^AdsA^, the ECF sigma factor required for aerial mycelium formation, and *amfR*, which encodes a protein essential for aerial mycelium formation, and AdpA can also regulate chromosome replication by binding to the region close to OriC [1, 2, 19, 20]. On the other hand, AdpA_*Sx*_ from *Streptomyces* *xiamenensis* 318 was reported to negatively regulate morphological differentiation [3]. At the same time, AdpA is capable of influencing the transcription of secondary metabolism genes both directly [16, 21, 22] and indirectly [11, 17, 18, 23–27] and was used to successfully activate secondary metabolism. The expression of *adpA* is subject to a noteworthy regulation of the translation level for the UUA codon within the *adpA* transcript, in addition to being controlled by the repressor protein ArpA, the hormone-like molecule and asRNA [7]. The UUA codon is the rarest codon in the GC-rich genomes of *Actinobacteria* and is present in approximately 2–3% of gene transcripts in any one *Streptomyces*, typically, based on the information from four sequenced genomes [28, 29]. The tRNA^Leu^_UAA_ that is capable of reading the UUA codon efficiently is only encoded by *bldA*, and its delayed occurrence limits the expression of TTA-containing genes during the cell cycle [14, 28, 30].

The extracellular metabolite ε-poly-l-lysine (ε-PL) is a kind of amino acid homopolymer composed of 25–35 l-lysine residues with isopeptide bonds between the α-carboxyl and ε-amino groups and is mainly produced by the *Streptomycetaceae* family [31–34]. ε-PL exhibits antimicrobial effects against a broad spectrum of microorganisms, including bacteria, yeasts, molds and some viruses, due to its polycationic nature [31, 35]. The desirable properties of ε-PL, including its water solubility, thermal stability, biodegradability, broad-spectrum antimicrobial activity and nontoxicity to humans and the environment, make it suitable for use in the cosmetic, pharmaceutical and electronics industries, and it is especially widely applied as a natural and safe food preservative in Korea, Japan, the USA, China and other countries [36, 37]. Moreover, ε-PL can also be applied in weight loss products and health care products and can be used as a drug carrier, gene carrier, biochip, bioelectronic coating agent and new water absorbent material [38, 39].

Although ε-PL has been industrially produced, the low fermentation efficiency and high cost still need to be resolved [40]. The nutrition feeding [41], medium optimization [42], dissolved oxygen regulation [43], pH control strategy [44, 45], solid-state fermentation [46], in situ product removal fermentation [47], fermentation with immobilized cells [48], atmospheric and room temperature plasma (ARTP) mutagenesis [49], genome shuffling [49], ribosome engineering [49–51] and molecular biology operations in regard to *vgb* (the *Vitreoscilla* hemoglobin gene) [52, 53], *amtB* (the ammonium transporter gene) [54], *pls* (the ε-PL synthetase gene) [39, 55], and *dapA* (the dihydrodipicolinate synthase gene) [56] have been developed to increase the yield of ε-PL. It is recognized that the precise controls of primary and secondary metabolism development and their switch are pivotal to properly produce invaluable natural products in *Streptomyces*, but few studies have focused on increasing the production of ε-PL effectively by manipulating transcription factors in *Streptomyces* thus far.

In this work, we revealed the regulatory roles of *Streptomyces* AdpA homologs in ε-PL biosynthesis and morphological differentiation and effectively promoted ε-PL production and sporulation in *S.* *albulus* NK660 by sieving out AdpA_*Sn*_ from five AdpA homologs, including the novel AdpA homolog (AdpA_*Sa*_) identified here. Subsequently, we clarified the mechanism by which AdpA_*Sn*_ affected ε-PL biosynthesis and its effect on the ε-PL polymerization degree in *S.* *albulus*. Furthermore, we identified seven target genes directly regulated by AdpA_*Sn*_ in the central metabolic pathways and acetate metabolism pathway of *S.* *albulus* NK660. We hope this study will provide a reference for the enhanced production of ε-PL and other valuable secondary metabolites in *Streptomyces*.

## Materials and methods

### Strains, plasmids, and culture conditions

All the bacterial strains and plasmids used in this study are listed in Table 1, and the primers are listed in Table S1. The culture conditions of *S.* *albulus* NK660 and its derivatives were as described previously [52]. *Escherichia* *coli* DH5α and *E.* *coli* BL21 (DE3) were used as the cloning host and the expression host, respectively. We used *E.* *coli* WM6026 [57] as a nonmethylating plasmid donor strain to perform intergeneric conjugation with *S.* *albulus* NK660. *E.* *coli* strains were grown in Luria–Bertani medium at 37 °C. When necessary, 30 μg/mL apramycin, 50 μg/mL kanamycin and 19 μg/mL diaminopimelic acid were added.

**Table 1:** Strains and plasmids used in this study

Strain or plasmid	Characteristics	Source or reference
Strains		
*S.* *albulus*		
NK660	Wild type (WT), ε-poly-l-lysine producer	[34]
SET	Wild-type strain carrying pSET152	This work
NKA	Wild-type strain carrying pSET152-*adpA*_*Sa*_	This work
SDA	Wild-type strain carrying pSET152-*adpA*_*Sd*_	This work
SHA	Wild-type strain carrying pSET152-*adpA-SH*	This work
SNA	Wild-type strain carrying pSET152-*adpA*_*Sn*_	This work
SCA	Wild-type strain carrying pSET152-*adpA-C*	This work
*E.* *coli*		
DH5α	General cloning host	TaKaRa
BL21(DE3)	Host for expression of AdpA_*Sn*_	TransGen Biotech
WM6026	Donor strain for conjugation between *E.* *coli* and *Streptomyces*	[57]
Plasmids		
pSET152	Integrative vector based on ϕC31 integrase	[58]
pSET152-*adpA*_*Sa*_	pSET152 derivative for overexpression of *adpA*_*Sa*_	This work
pSET152-*adpA*_*Sd*_	pSET152 derivative for expression of *adpA*_*Sd*_	This work
pSET152-*adpA-SH*	pSET152 derivative for expression of *adpA-SH*	This work
pSET152-*adpA*_*Sn*_	pSET152 derivative for expression of *adpA*_*Sn*_	This work
pSET152-*adpA-C*	pSET152 derivative for expression of *adpA-C*	This work
pET-28a ( +)	*E.* *coli* expression vector	Novagen
pET-SNA	*adpA*_*Sn*_ cloned in pET-28a ( +)	This work

### Construction of *S. albulus* mutant strains

For overexpression of *adpA*_*Sa*_, the DNA fragment containing the *adpA*_*Sa*_ encoding region was amplified from the genome DNA of *S.* *albulus* NK660 with the primer pair *adpA*-F/-R. This DNA fragment was joined with the synthesized promoter PermE* (GenScript, Nanjing, China) by overlap-PCR. Under the catalysis of Exnase II (Vazyme, Nanjing, China), the generated DNA fragment was ligated into the vector pSET152 [58] that was digested with XbaI to generate the *adpA*_*Sa*_ overexpression vector pSET152-*adpA*_*Sa*_, which was then transformed into *S.* *albulus* NK660 to construct the *adpA*_*Sa*_ overexpression strain *S.* *albulus* NKA. To construct the heterogeneous expression vectors, DNA fragments containing the promoter PermE* and one of the genes encoding AdpA_*Sd*_ (AFX97763.1), AdpA-SH (WP_018531726.1), AdpA_*Sn*_ (WP_055538474.1) or AdpA-C (WP_007264197.1) were synthesized (GenScript, Nanjing, China). Similarly, the obtained DNA fragments were ligated into the integrative vector pSET152 digested with XbaI by Exnase II (Vazyme, Nanjing, China), successively generating the vectors pSET152-*adpA*_*Sd,*_ pSET152-*adpA-SH,* pSET152-*adpA*_*Sn*_ and pSET152-*adpA-C*, which were then transformed into *S.* *albulus* NK660 to construct the heterogenous overexpression strains *S.* *albulus* SDA, *S.* *albulus* SHA, *S.* *albulus* SNA and *S.* *albulus* SCA*.* Furthermore, to investigate the effect of pSET152, the empty plasmid pSET152 was also integrated into the *S.* *albulus* NK660 genome, generating *S.* *albulus* SET. All the constructed mutants were verified by PCR and DNA sequencing with the primer pair SET-F/-R. DNA manipulations were carried out using standard procedures for *Streptomyces* and *E.* *coli* [59, 60].

### RNA sample preparation and RT–PCR, and RT–qPCR analysis

Total mRNA was isolated from *S.* *albulus* NK660 and its derivatives after 29 h of growth in M3G medium using a SPARKeasy Improved Bacteria RNA kit according to the manufacturer’s protocol (SparkJade, Shandong, China). Isolated RNA was reverse transcribed using HiScript^®^ II Reverse Transcriptase (Vazyme, Nanjing, China). For RT–PCR, the Genome DNA clearance column (a component of SPARKeasy Improved Bacteria RNA kit; SparkJade, Shandong, China)-treated RNA samples without reverse transcription were used as the negative controls to verify the absence of DNA contamination. The transcription levels of genes were determined by RT–qPCR (using the primer pairs listed in Additional file 1: Table S1) in triplicate for each transcript using ChamQ Universal SYBR qPCR Master Mix (Vazyme, Nanjing, China). The housekeeping gene *hrdB* was used for normalizing samples as the internal control with quantification by the 2^-∆∆CT^ method [61].

### Scanning electron microscopy

*S.* *albulus* NK660 and its derivatives grown on mannitol soya flour (MSF) agar plates at 30 °C were observed by scanning electron microscopy (SEM). For specimen preparation, coverslips were embedded in agar inoculated with the strains at an angle and lifted out gently after 2 or 3 days of incubation for follow-up operation. Samples were fixed with 2.5% glutaraldehyde solution overnight, washed three times with phosphate buffer, subsequently dehydrated by an ethanol concentration gradient (30%, 50%, 70%, 85%, 95% and 100%), freeze-dried (LGJ-12, SongYuanHuaXing), coated in gold, and then examined by SEM (MERLIN Compact, ZEISS).

### Flask culture and analytical procedures

*albulus* NK660 and its derivatives were inoculated in M3G medium at 30 °C with shaking (180 rpm) for 24 h as seed cultures, and then 5% (v/v) of the inocula were inoculated into M3G medium and cultured for 5 days at 30 °C with shaking (180 rpm). Precipitates of samples were harvested at specific time points and washed twice with distilled water. Then, they were dried by a freeze dryer (LGJ-12, SongYuanHuaXing) and subsequently weighed for the measurement of dry cell weights (DCWs). The supernatant of each sample was harvested at certain time points and used to determine the concentration of ε-PL on the basis of the procedures described by Itzhaki [52]. All cultivations were performed at least three times.

### Microscale thermophoresis assay

The DNA fragments corresponding to the promoter regions of genes *zwf,* *pyk2* and *pepc* were amplified and labeled using the FAM-labeled primer pairs *zwf*-F/-R, *pyk2*-F/-R and *pepc*-F/-R, and they were subsequently recovered by the Multifunctional DNA Purification and Recovery Kit (Aidlab, Beijing, China). The purified His-tagged AdpA_*Sn*_ (GenScript, Nanjing, China) was concentrated by centrifugal filters (Merck Milliproe, Germany). For each dissociation constant measurement, the 32 μM initial concentration of AdpA_*Sn*_ underwent a 16-step serial twofold dilution with PBS buffer, and the 16 diluted protein solutions were mixed with equal labeled DNA fragments. The mixtures were incubated for 30 min at 25 °C and transferred to standard Monolith NT.115 capillaries for follow-up measurements. The experiments were run at 40% excitation with high microscale thermophoresis (MST) power at room temperature on a Monolith NT.115 instrument (NanoTemper Technologies GmbH, Munich, Germany) [62]. The data were obtained using MO. Control 1.5.3 (NanoTemper Technologies GmbH, Munich, Germany) and analyzed using MO. Affinity Analysis 2.3 (NanoTemper Technologies GmbH, Munich, Germany).

### Molecular weight determination of ε-PL

The operations to separate and purify ε-PL from fermentation cultures were performed as described previously [34]. The purified ε-PL samples were analyzed using MALDI-TOF–MS with an Autoflex III TOF/TOF 200 (Bruker Corporation) instrument with α-cyano-4-hydroxycinnamic acid (CHCA) as the matrix [63].

### Multiple sequence alignment and phylogenetic analysis

The homologous sequence database search was performed using BLASTp (https://blast.ncbi.nlm.nih.gov/Blast.cgi). The multiple sequence alignment of the AdpA homologs was executed using the online available tool CLUSTALW (https://www.genome.jp/tools-bin/clustalw) and analyzed by ESPript 3.0 (http://espript.ibcp.fr/ESPript/cgi-bin/ESPript.cgi). Phylogenetic analyses were conducted in MEGA-X using the maximum parsimony method [64, 65].

## Results

### Identification of a novel AdpA homolog AdpA_***Sa***_ in ***S. albulus*** NK660

**Fig. 1:**
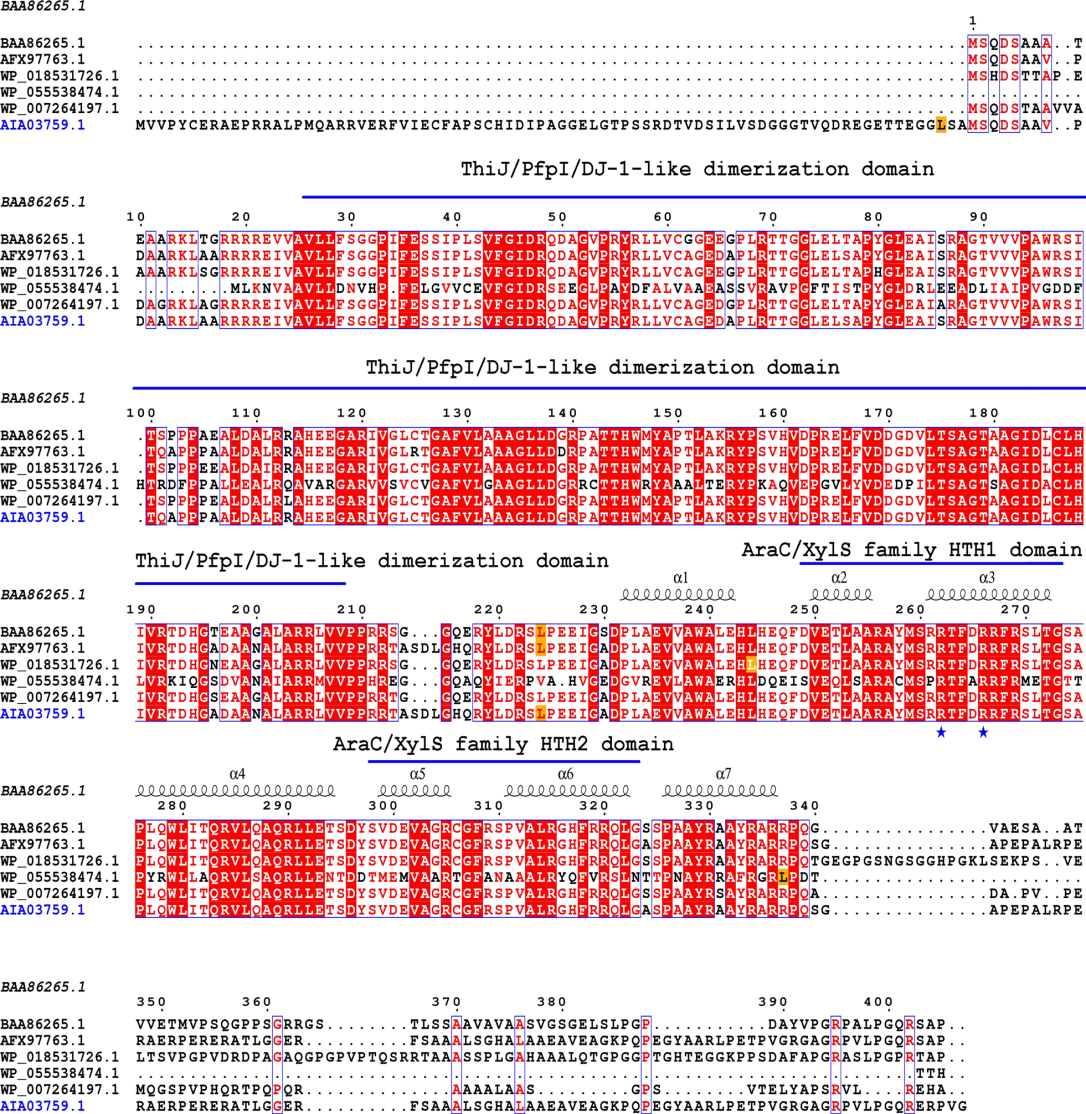
Amino acid alignment among AdpA_*Sa*_, AdpA_*Sd*_, AdpA-SH, AdpA_*Sn*_, AdpA-C and the well-characterized AdpA_*Sg*_. The ThiJ/PfpI/DJ-1 like domain for dimerization and two AraC/XylS family helix-turn-helix (HTH) motifs are marked. The locations of the Leu residues translated by the UUA codons are colored orange, and the locations of Arg-262 and Arg-266 residues that directly recognize the target DNA sequences of AdpA_*Sg*_ are marked with the blue stars. BAA86265.1, AdpA_*Sg*_; AFX97763.1, AdpA_*Sd*_; WP_018531726.1, AdpA-SH; WP_055538474.1, AdpA_*Sn*_; WP_007264197.1, AdpA-C; and the blue font AIA03759.1 is the endogenous AdpA_*Sa*_ in *S.* *albulus* NK660

We identified a novel AdpA homolog in *S.* *albulus* NK660 because the protein (GenBank accession no. AIA03759.1) shared 80% and 89% amino acid identities with the AdpA homologs of *S.* *griseus* and *S.* *coelicolor*, respectively, and designated it AdpA_*Sa*_. AdpA_*Sa*_ was found between the upstream gene encoding a putative universal stress protein and the downstream gene *ornA* that encodes a putative oligoribonuclease in *S.* *albulus* NK660 based on computer-assisted analysis (Additional file 1: Fig. S1A). This gene arrangement in *S.* *albulus* NK660 was similar to those found in *S.* *griseus*, *Streptomyces* *coelicolor,* *Streptomyces* *chattanoogensis* and *Streptomyces* *clavuligerus* (Additional file 1: Fig. S1A) [17, 25, 66]. Similar to most AdpA orthologous proteins, AdpA_*Sa*_ possessed a ThiJ/pfpI/DJ-1-like domain for dimerization and a DNA-binding domain with two AraC/XylS family helix-turn-helix motifs (Fig. 1). It was reported that the Arg262 and Arg266 residues of AdpA_*Sg*_ could directly recognize the target DNA sequences of AdpA_*Sg*_ [67], and AdpA_*Sa*_ retained these two arginine residues at the corresponding positions of the HTH1 motif (Fig. 1). Different from most AdpA proteins, there were two UUA codons in *adpA*_*Sa*_, and they were located in front of the ThiJ/PfpI/DJ-1-like dimerization domain and at the ʻclassicalʼ position between the ThiJ/pfpI/DJ-1-like domain and HTH domains (Fig. 1), indicating that the translation level of *adpA*_*Sa*_ could be affected by *bldA* [7].

To show the relationship between AdpA_*Sa*_ and other AdpA proteins, a phylogenetic tree that was divided into three clades was constructed, and AdpA_*Sa*_ was found in clade I (Additional file 1: Fig. S1B). AdpA_*Sd*_ was reported to positively regulate toyocamycin biosynthesis and morphological differentiation in *Streptomyces* *diastatochromogenes* 1628 [68], and it was found in the same subclade as AdpA_*Sa*_ (Additional file 1: Fig. S1B), which indicates that AdpA_*Sa*_ may play a similar role in morphological differentiation and secondary metabolism in *S.* *albulus* NK660.

### AdpA_*Sa*_ is an activator of both morphological differentiation and ε-PL production in *S. albulus*

To study the regulatory role of AdpA_*Sa*_ in *S.* *albulus*, we constructed an *adpA*_*Sa*_ overexpression strain, *S.* *albulus* NKA (Table 1 and Additional file 1: Fig. S2A). The construction of *S.* *albulus* NKA was further confirmed by PCR (Additional file 1: Fig. S2B) and DNA sequencing. RT–qPCR analysis showed that the *adpA*_*Sa*_ transcription level in *S.* *albulus* NKA was approximately sixfold that in WT, indicating that *adpA*_*Sa*_ was successfully overexpressed in *S.* *albulus* NKA (Additional file 1: Fig. S2C).

**Fig. 2:**
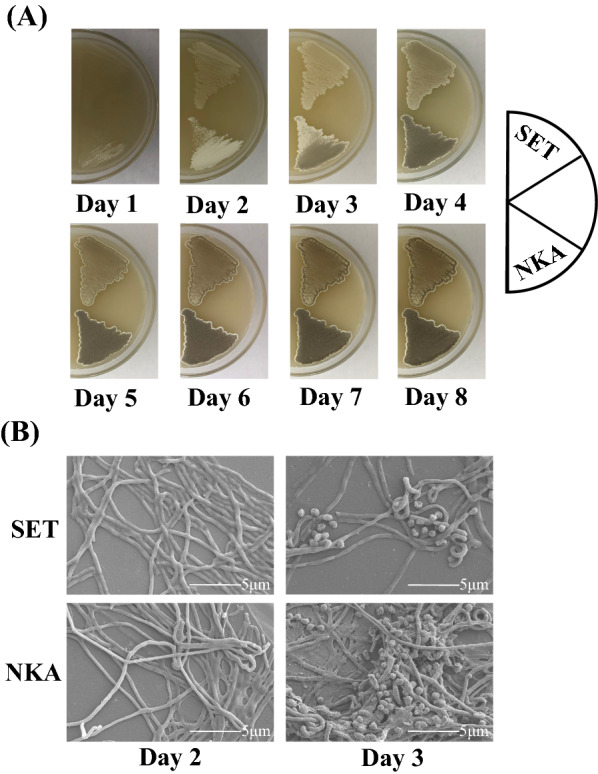
Effects of AdpA_*Sa*_ on morphological development in *S.* *albulus*. **A** Phenotypes of *S.* *albulus* SET and *S.* *albulus* NKA grown on MSF agar plates at 30 ℃. **B** SEM showing morphological development of *S.* *albulus* SET and *S.* *albulus* NKA grown on MSF agar plates

To clarify the effect of AdpA_*Sa*_ on development in *S.* *albulus*, the aerial mycelium formation and sporulation of *S.* *albulus* NKA were compared with those of the control strain *S.* *albulus* SET by plate streaking and SEM. As shown in Fig. 2, the *S.* *albulus* NKA more abundantly sporulated than *S.* *albulus* SET, of which colonies on Day 3 consisted of only aerial hyphae and a few spores, while colonies of *S.* *albulus* NKA had already made a large amount of mature spores. These findings indicate that AdpA_*Sa*_ acts as an activator of development in *S.* *albulus,* which is similar to the function of AdpA_ch_ in *S.* *chattanoogensis* [17], indicating that AdpA homologs play a primary role in the morphological differentiation of *Streptomyces*.

**Fig. 3:**
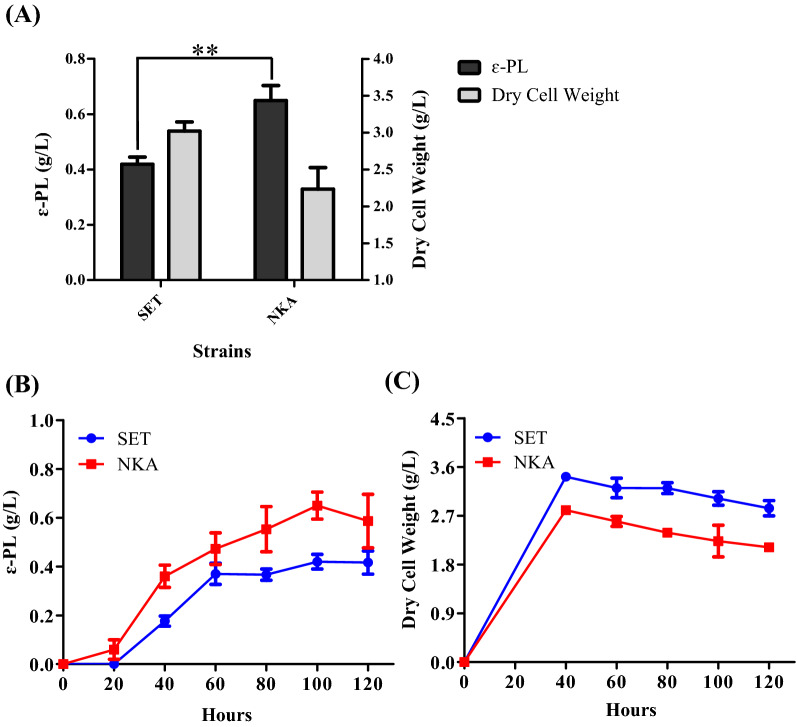
Effects of AdpA_*Sa*_ on ε-PL production and cell growth in *S.* *albulus*. **A** ε-PL yield and dry cell weight in *S.* *albulus* NKA and *S.* *albulus* SET cultured in M3G medium for 100 h. **P < 0.01 (Student's *t*-test). **B** Time course of ε-PL yield in *S.* *albulus* SET and *S.* *albulus* NKA cultured in M3G medium. **C** Growth curves (biomass presented by dry cell weight) of *S.* *albulus* SET and *S.* *albulus* NKA cultured in M3G medium

To investigate the effect of AdpA_*Sa*_ on ε-PL biosynthesis in *S.* *albulus*, we performed flask-shaking fermentation between *S.* *albulus* NKA and *S.* *albulus* SET and finally measured their own ε-PL production and biomass (DCW) separately. On the one hand, when cultured for 100 h, *S.* *albulus* NKA (0.65 g/L, 2.96 mg g^−1^ h^−1^) separately had a 1.5-fold and 2.1-fold increase in ε-PL production and specific ε-PL formation rates compared with *S.* *albulus* SET (0.42 g/L, 1.39 mg g^−1^ h^−1^) (Fig. 3A and Additional file 1: Fig. S2D), which indicated that AdpA_*Sa*_ positively regulated ε-PL production in *S.* *albulus*. On the other hand, the biomass of *S.* *albulus* NKA was 26.2% lower than that of *S.* *albulus* SET according to the results shown in Fig. 3A.

Therefore, to further clarify the specific process by which the biomass and ε-PL of *S.* *albulus* SET and *S.* *albulus* NKA accumulated, we measured the time courses of biomass and ε-PL yield in these two strains. The results showed that AdpA_*Sa*_ promoted the production of ε-PL in *S.* *albulus* from several aspects. First, because of the same fermentation time (100 h) required for *S.* *albulus* SET and *S.* *albulus* NKA to obtain their own peak ε-PL production and the fact that the overexpression of *adpA*_*Sa*_ caused an earlier time to start producing ε-PL (Fig. 3B), the overexpression of *adpA*_*Sa*_ could prolong the total time that ε-PL was accumulated even though the actual total time of fermentation did not change. Second, the overexpression of *adpA*_*Sa*_ might solve some rate-limiting problems of ε-PL production in *S.* *albulus*. This is because *S.* *albulus* NKA missed the rate-limiting period in which ε-PL accumulated slowly and maintained a sustained increase in ε-PL yield during the 100-h culture in fermentation medium, unlike *S.* *albulus* SET (Fig. 3B). Third, the ε-PL yield of *S.* *albulus* NKA remained above that of *S.* *albulus* SET during the whole fermentation process (Fig. 3B), indicating that AdpA_*Sa*_ was capable of enhancing the ability to produce ε-PL in *S.* *albulus* during the whole fermentation process. All these regulatory functions of AdpA_*Sa*_ were collectively taken together to promote the production of ε-PL in *S.* *albulus*. However, as shown in Fig. 3C, the biomass of *S.* *albulus* SET remained higher than that of *S.* *albulus* NKA during the whole fermentation process.

In conclusion, AdpA_*Sa*_ was identified as a pleiotropic regulator involved in morphological development, secondary metabolism and growth of *Streptomyces.* AdpA_*Sa*_ acts as an activator for sporulation and ε-PL biosynthesis in *S.* *albulus*, which agrees with the conclusion drawn from the phylogenetic analysis of AdpA_*Sa*_ before (Additional file 1: Fig. S1B).

### Molecular sequences and phylogenetic relationships of *adpA* genes

Because AdpA_*Sa*_ is an activator for morphological differentiation and ε-PL biosynthesis in *S.* *albulus,* we expected to further promote morphological differentiation and obtain a higher ε-PL production by the expression of suitable heterologous *adpA* in *S.* *albulus.* Considering that the expression of *adpA* is restricted to the level at which the rare UUA codon within the *adpA* transcript is translated to leucyl, four *adpA* genes were selected to be heterologously expressed in *S.* *albulus* NK660 according to their diversities of the number and position of UUA codons. These four *adpA* genes were separate from *S.* *diastatochromogenes* 1628 (*adpA*_*Sd*_), *Streptomyces* sp. HmicA12 (*adpA-SH*), *Streptomyces* *neyagawaensis* NRRLB-3092 (*adpA*_*Sn*_) and *Streptomyces* sp. C (*adpA-C*). Similar to the UUA codon of *adpA*_*Sg*_, the UUA codon of *adpA*_*Sd*_ is located in the ʻclassicalʼ position between the ThiJ/PfpI/DJ-1-like dimerization domain and HTH domains (Fig. 1). The UUA codon of *adpA-SH* is near the beginning of the AraC/XylS-type DNA-binding domain, and that of *adpA*_*Sn*_ is located close to the stop codon, while *adpA-C* has no UUA codon at all (Fig. 1). Identical to endogenous AdpA_*Sa*_, the four heterologous AdpA proteins studied in this paper also have a ThiJ/pfpI/DJ-1-like domain for dimerization and a DNA-binding domain with two AraC/XylS family helix-turn-helix motifs (Fig. 1). Moreover, these four AdpA proteins all retain the two arginine residues at the positions corresponding to the AdpA_*Sg*_ Arg-262 and Arg-266 residues that directly recognize the target DNA sequences of AdpA_*Sg*_ (Fig. 1) [67]. As mentioned above, AdpA_*Sd*_ was found in clade I of the phylogenetic tree, similar to AdpA_*Sa*_ (Additional file 1: Fig. S1B). As the regulatory roles of AdpA_*Sd*_ in *S.* *diastatochromogenes* 1628 were reported before [68], we expected to let AdpA_*Sd*_ promote morphological development and secondary metabolism in *S.* *albulus* by heterologously expressing AdpA_*Sd*_. The other three heterologous AdpA proteins that we studied were all located in clade II. AdpA-SH and AdpA-C belonged to subclade II-a, while AdpA_*Sn*_ belonged to subclade II-b.

The *adpA*_*Sd*_, *adpA-SH*, *adpA*_*Sn*_ and *adpA-C* genes were placed under the control of the strong promoter PermE*, and the heterogeneous overexpression strains *S.* *albulus* SDA, *S.* *albulus* SHA, *S.* *albulus* SNA and *S.* *albulus* SCA were constructed (Additional file 1: Fig. S3A). The PCR (Additional file 1: Fig. S3B) and DNA sequencing results confirmed that these heterologous *adpA* genes were successfully inserted into *S.* *albulus* NK660 separately. As shown in Additional file 1: Fig. S3C, the results of RT–PCR between the four mutants expressing heterologous *adpA* genes and the control strains showed that these four heterologous *adpA* genes were successfully transcribed in mutants separately.

### AdpA_*Sn*_ is the strongest activator of both ε-PL biosynthesis and morphological differentiation among all five AdpA homologs

**Fig. 4:**
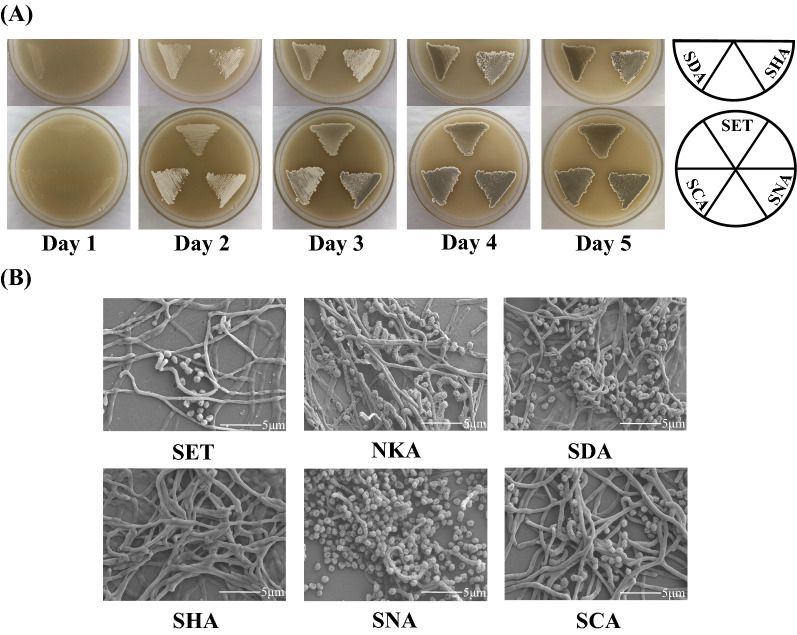
Effects of AdpA_*Sd*_, AdpA-SH, AdpA_*Sn*_ and AdpA-C on morphological development in *S.* *albulus*. **A** Phenotypes of four heterologous *adpA* genes expression mutants and *S.* *albulus* SET grown on MSF agar plates at 30 ℃. **B** SEM showing morphological development of four heterologous *adpA* genes expression mutants, *S.* *albulus* NKA and *S.* *albulus* SET grown on MSF agar plates for 3 days

To clarify the influences of these four heterologous *adpA* genes on development in *S.* *albulus*, plate streaking and SEM between the four mutants expressing heterologous *adpA* genes and the control strain *S.* *albulus* SET were performed. On the one hand, in comparison with *S.* *albulus* SET, the mutants *S.* *albulus* SDA, *S.* *albulus* SNA and *S.* *albulus* SCA more abundantly sporulated (Fig. 4). When incubated for 3 days, these three mutants had already developed many mature spores, whereas *S.* *albulus* SET produced only aerial hyphae and a small quantity of spores (Fig. 4B). On the other hand, heterologous expression of the *adpA-SH* gene caused considerably delayed spore formation in *S.* *albulus* (Fig. 4). The colonies of *S.* *albulus* SHA on Day 3 consisted of almost pure aerial hyphae, and there were no spores or sporulation septa that could be observed (Fig. 4B). Among all the mutants, the *S.* *albulus* SNA strain most abundantly sporulated, and its colonies consisted of almost entirely mature spores when incubated for 3 days (Fig. 4B).

**Fig. 5:**
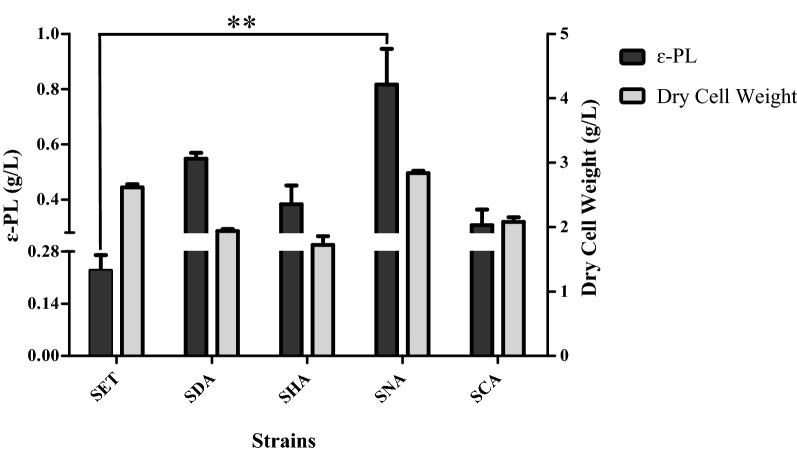
The ε-PL yield and dry cell weight in four heterologous *adpA* genes expression mutants and *S.* *albulus* SET cultured in M3G medium. **P < 0.01 (Student's *t*-test)

To further investigate the effects of the four heterologous *adpA* genes on ε-PL biosynthesis in *S.* *albulus*, flask-shaking fermentation between the four mutants expressing heterologous *adpA* genes and the control strain *S.* *albulus* SET was performed. An expected result shown in Fig. 5 is that all four mutants expressing heterologous *adpA* genes obtained higher ε-PL yields than *S.* *albulus* SET to different degrees separately. Among the four mutants expressing heterologous *adpA* genes, the strain *S.* *albulus* SNA obtained the highest ε-PL yield of 0.82 g/L, which was approximately 3.6-fold that in *S.* *albulus* SET (Fig. 5). *S.* *albulus* SDA, *S.* *albulus* SHA and *S.* *albulus* SCA resulted in higher ε-PL yields than *S.* *albulus* SET, with increases of 139.1% (0.55 g/L), 65.2% (0.38 g/L) and 34.8% (0.31 g/L), respectively (Fig. 5). Among the four mutants expressing heterologous *adpA* genes, the strain *S.* *albulus* SNA obtained the highest biomass of 2.84 g/L, which was similar to that of *S.* *albulus* SET, while the other three mutants *S.* *albulus* SDA, *S.* *albulus* SHA and *S.* *albulus* SCA exhibited 26.0%, 34.4%, and 20.6% declines in biomass compared with *S.* *albulus* SET, respectively (Fig. 5). These results indicate that the *adpA*_*Sn*_ gene was the only gene that promoted ε-PL production without any adverse impact on growth in *S.* *albulus* among all the *adpA* genes studied in this paper.

In conclusion, the above results confirm that AdpA-SH functions as a repressor of development but an activator of ε-PL production in *S.* *albulus*, whereas AdpA_*Sd*_, AdpA_*Sn*_ and AdpA-C act as activators both for sporulation and ε-PL biosynthesis in *S.* *albulus*, which is similar to the function of endogenous AdpA_*Sa*_ in *S.* *albulus* NK660. It is worth mentioning that the heterologous AdpA_*Sn*_ has the strongest abilities to positively regulate both morphological differentiation and ε-PL biosynthesis without any adverse impact on growth in *S.* *albulus* among all the AdpA homologs studied in this paper.

### AdpA_*Sn*_ promotes ε-PL production by affecting the transcription of key genes in ε-PL biosynthesis

**Fig. 6:**
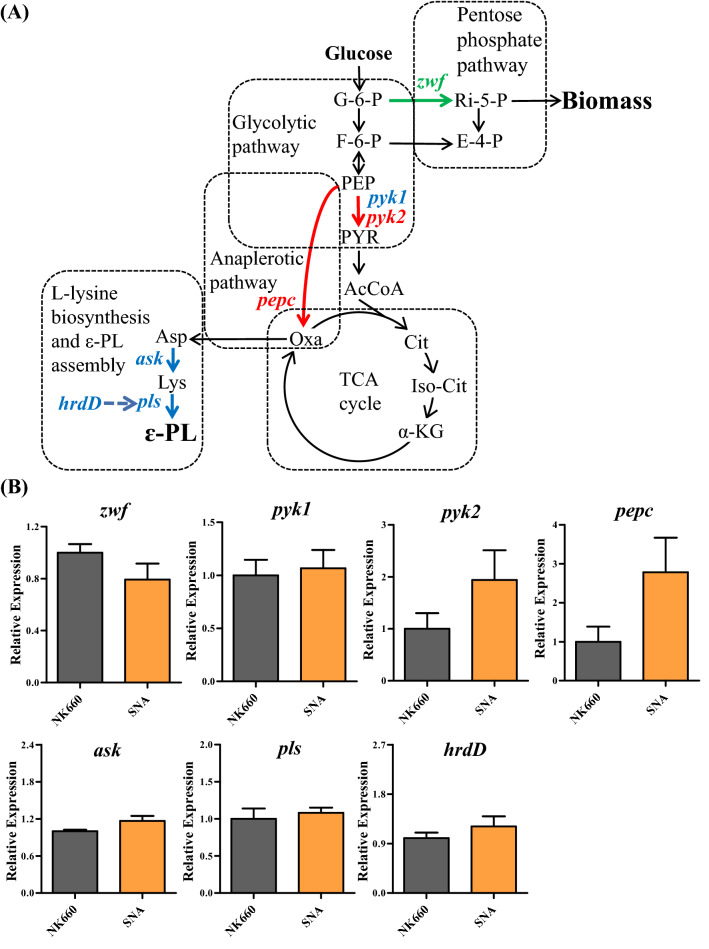
Simplified metabolic network and transcription levels of key genes for ε-PL biosynthesis in *S.* *albulus.* **A** Simplified metabolic network of ε-PL production using glucose as the carbon source in *S.* *albulus*. The key genes for ε-PL biosynthesis are indicated, and the green, red and blue fonts or arrows stand for decreased transcription level, increased transcription level and no significant change in transcription level of these genes caused by the expression of *adpA*_*Sn*_, respectively. The dotted arrow means the transcription level of *pls* is regulated by the sigma factor HrdD. **B** Transcription levels of the key genes for ε-PL biosynthesis in *S.* *albulus* SNA and *S.* *albulus* NK660

To further investigate the specific effect of AdpA_*Sn*_ on ε-PL production in *S.* *albulus*, the transcription levels of seven key genes in ε-PL biosynthesis were measured. ε-PL biosynthesis involves the glycolytic pathway (EMP), pentose phosphate pathway (PPP), anaplerotic metabolic pathway, tricarboxylic acid (TCA) cycle, diaminopimelic acid pathway (DAP) and ε-PL assembly in *Streptomyces* (Fig. 6A) [51, 69, 70]. Glucose-6-phosphate dehydrogenase (G6PDH) is the rate-limiting enzyme of the pentose phosphate pathway that is responsible for providing NADPH and pentoses used for cell growth as well as metabolite biosynthesis [70–72]. The fact that the transcription level of *zwf* (the only gene encoding G6PDH in *S.* *albulus* NK660) in *S.* *albulus* SNA was 20.8% lower than that in *S.* *albulus* NK660 (Fig. 6B) might cause a metabolic shift, which indicates that more carbon skeletons derived from glucose were used for glycolysis (EMP) than for cell growth (PPP) in comparison with *S.* *albulus* NK660. As an important role in carbon metabolism, pyruvate kinase (PK) links glycolysis, gluconeogenesis and the TCA cycle and plays a central part in the generation of adenosine triphosphate (ATP) and precursors for specialized metabolites such as acetyl-CoA, amino acids, and organic acids [73]. There are two pyruvate kinases that are encoded by the genes *pyk1* and *pyk2* in *S.* *albulus* NK660. As shown in Fig. 6B, the transcription level of *pyk2* in *S.* *albulus* SNA was 1.9-fold that in *S.* *albulus* NK660, while no distinct difference was found in the transcription level of *pyk1* between *S.* *albulus* SNA and *S.* *albulus* NK660, indicating that a larger amount of the direct precursor for acetyl-CoA and more ATP might be produced by the heterologous expression of the *adpA*_*sn*_ gene. As one of the enzymes included in the phosphoenolpyruvate-pyruvate-oxaloacetate node, which is a major branch in central carbon metabolism, phosphoenolpyruvate carboxylase (PEPC) is capable of catalyzing the carboxylation of phosphoenolpyruvate to generate oxaloacetate for the TCA cycle and other metabolic processes necessary for growth [74]. The transcription level of *pepc* in *S.* *albulus* SNA was 2.8-fold higher than that in *S.* *albulus* NK660 (Fig. 6B), indicating that AdpA_*Sn*_ might prompt the replenishment of more TCA cycle intermediates for L-lysine biosynthesis and cause the generation of more carbon skeletons and ATP for ε-PL biosynthesis by affecting the transcription of *pepc*. ε-PL biosynthesis is catalyzed by Pls with ATP and the precursor l-lysine consumption [75], and aspartokinase (ASK) plays a key role in the biosynthesis of l-lysine [70]. Pls is a membrane protein that directly generates ε-PL chain length diversity [76], and the expression of *pls* is regulated by the sigma factor HrdD, which initiates ε-PL biosynthesis in response to variations in pH in vivo [77]. The transcription of *ask*, *pls* and *hrdD* showed no significant difference between *S.* *albulus* SNA and *S.* *albulus* NK660 (Fig. 6B). Taken together, these results demonstrated that AdpA_*Sn*_ might cause the redistribution of metabolic flux in central metabolism pathways by affecting the transcription of *zwf,* *pyk2* and *pepc*, which subsequently produced more carbon skeletons and ATP for ε-PL biosynthesis in *S.* *albulus*.

### AdpA_*Sn*_ directly regulates the *zwf*, *pyk2* and *pepc* genes

**Fig. 7:**
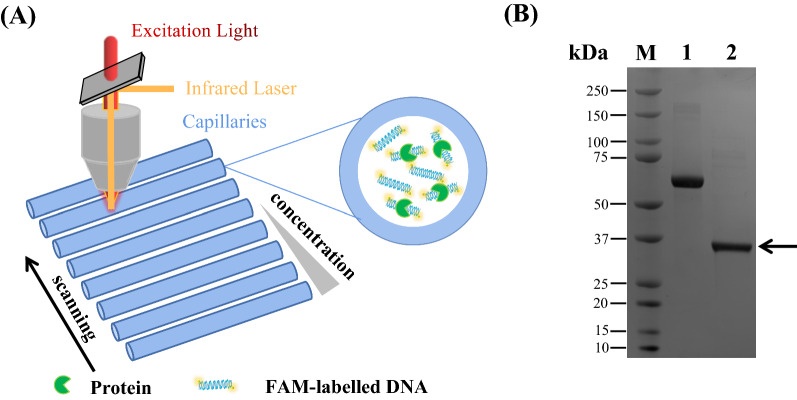
Schematic diagram and purified AdpA_*Sn*_ protein for MST analysis. **A** Schematic diagram of the MST analysis. **B** SDS-PAGE of the purified AdpA_*Sn*_ protein for MST analysis. Lane M, protein marker; Lane 1, bovine serum albumin (BSA); Lane 2, purified His-tagged AdpA_*Sn*_ protein

**Fig. 8:**
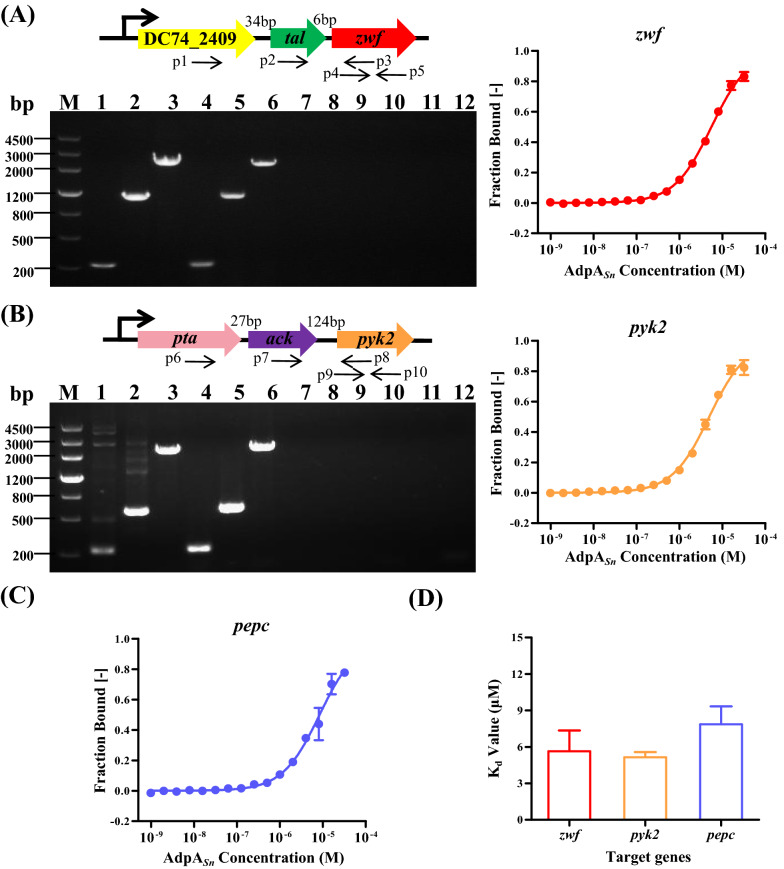
Interaction between promoter regions of targets and AdpA_*Sn*_. **A** Interaction between promoter region of *zwf* and AdpA_*Sn*_. The upstream genes of *zwf*, intergenic distances among these three genes and their common promoter region are displayed. RT-PCR results confirmed that genes DC74_2409, *tal* and *zwf* shared common promoter: Lane M, DNA marker III; Lane 1,4,7,10, amplification products using primers p4/p5 with gDNA, cDNA, RNA from *S.* *albulus* SNA and ddH_2_O as templates, respectively; Lane 2,5,8,11, amplification products using primers p2/p3 with gDNA, cDNA, RNA from *S.* *albulus* SNA and ddH_2_O as templates, respectively; Lane 3,6,9,12, amplification products using primers p1/p3 with gDNA, cDNA, RNA from *S.* *albulus* SNA and ddH_2_O as templates, respectively. The binding curve for interaction between promoter region of *zwf* and AdpA_*Sn*_ is indicated. **B** Interaction between promoter region of *pyk2* and AdpA_*Sn*_. The upstream genes of *pyk2*, intergenic distances among these three genes and their common promoter region are displayed. RT-PCR results confirmed that genes *pta*, *ack* and *pyk2* shared common promoter: Lane M, DNA marker III; Lane 1,4,7,10, amplification products using primers p9/p10 with gDNA, cDNA, RNA from *S.* *albulus* SNA and ddH_2_O as templates, respectively; Lane 2,5,8,11, amplification products using primers p7/p8 with gDNA, cDNA, RNA from *S.* *albulus* SNA and ddH_2_O as templates, respectively; Lane 3,6,9,12, amplification products using primers p6/p8 with gDNA, cDNA, RNA from *S.* *albulus* SNA and ddH_2_O as templates, respectively. The binding curve for interaction between promoter region of *pyk2* and AdpA_*Sn*_ is indicated. **C** The binding curve for interaction between promoter region of *pepc* and AdpA_*Sn*_. **D** Comparison of the binding affinities between AdpA_*Sn*_ and the promoter regions of three target genes

To determine whether AdpA_*Sn*_ regulates the transcription of genes *zwf,* *pyk2* and *pepc* directly or through other regulators, the binding affinities of purified His-tagged AdpA_*Sn*_ for the promoter regions of *zwf,* *pyk2* and *pepc* were measured via MST that is based on thermophoresis, the directional movement of molecules in the temperature gradient (Fig. 7) [78]. As shown in Fig. 8A, the genes encoding transketolase (DC74_2409) and transaldolase (*tal*, DC74_2410) are cotranscribed with *zwf*, and they are all under the control of the promoter located in front of the gene encoding transketolase. The upstream genes encoding phosphate acetyltransferase (*pta*, DC74_5557) and acetate kinase (*ack*, DC74_5556) are cotranscribed with *pyk2*, and these three genes share a common promoter (Fig. 8B). The MST results showed that AdpA_*Sn*_ was able to directly bind to the promoter regions of z*wf,* *pyk2* and *pepc* with dissociation constants (*K*_d_) of 5.5 ± 0.3 µM, 4.9 ± 0.5 µM and 8.4 ± 0.9 µM, respectively (Fig. 8). We measured the binding curves between the same concentration of BSA as AdpA and the studied DNA fragments as the negative controls, and no specific bindings to the DNA probes were detected.

### Effect of AdpA_*Sn*_ on the polymerization degree of ε-PL

**Fig. 9:**
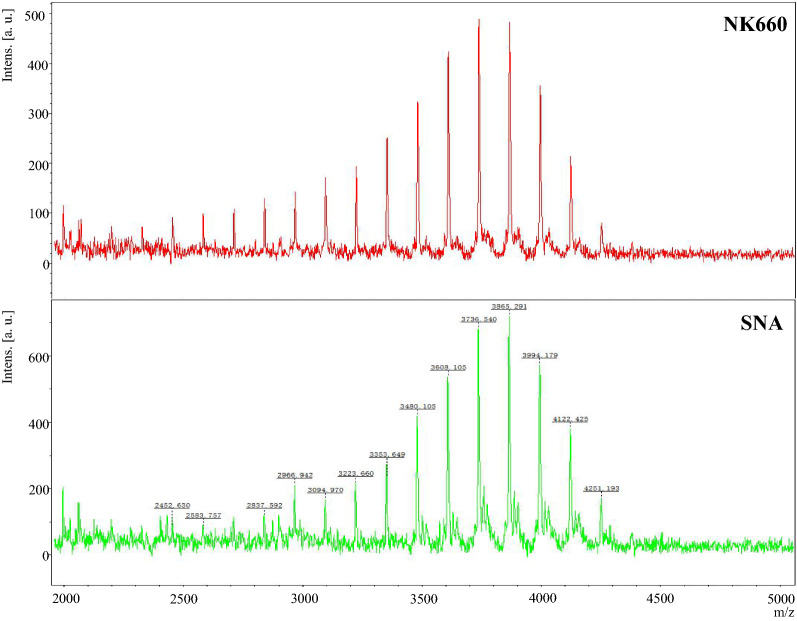
Polymerization degree of the purified ε-PL products produced by *S.* *albulus* NK660 and *S.* *albulus* SNA, respectively

To elucidate whether AdpA_*Sn*_ had any impact on the ε-PL polymerization degree in *S.* *albulus*, the relative molecular mass distributions of the ε-PL produced by *S.* *albulus* SNA and *S.* *albulus* NK660 were determined by MALDI-TOF–MS. As shown in Fig. 9, the molecular mass distribution of the ε-PL produced by *S.* *albulus* SNA ranged from 2453 to 4251 Da, corresponding to the polymerization degree of 19–33 L-lysine monomers, which was identical to that of *S.* *albulus* NK660. These results indicate that AdpA_*Sn*_ did not affect the polymerization degree of ε-PL in *S.* *albulus.*

## Discussion

In general, morphological differentiation and secondary metabolism in *Streptomyces* are regulated by complex regulatory networks in response to numerous environments and growth conditions [3]. It was reported that Pls catalyzes l-lysine polymerization to generate ε-PL and that the expression of *pls* was regulated by the sigma factor HrdD, whereas other regulatory effects involved in ε-PL biosynthesis are still unknown. In this study, we identified a novel AdpA homolog named AdpA_*Sa*_ in *S.* *albulus* NK660 and investigated the regulatory effects of endogenous AdpA_*Sa*_ and four heterologous AdpA proteins on ε-PL biosynthesis and morphological differentiation in *S.* *albulus*. On the one hand, all five mutants separately integrated with different *adpA* genes produced more ε-PL than the control strain *S.* *albulus* SET, which indicated that they all acted as activators for ε-PL biosynthesis in *S.* *albulus*, and the positive regulatory roles of *Streptomyces* AdpA homologs in ε-PL biosynthesis were revealed for the first time. On the other hand, the effects of AdpA homologs on morphological differentiation are diversified. Similar to the function of endogenous AdpA_*Sa*_, AdpA_*Sd*_, AdpA_*Sn*_ and AdpA-C act as activators of sporulation in *S.* *albulus*, while AdpA-SH functions as a repressor of development. Therefore, AdpA-SH becomes the second one found to have a negative effect on morphological differentiation in addition to AdpA_*Sx*_ from *S.* *xiamenensis* 318 [3], which indicates that our research provides additional evidence for the diversified regulation of AdpA homologs in morphological differentiation. It is worth mentioning that the heterologous AdpA_*Sn*_ possessed the strongest abilities to positively regulate both morphological differentiation and ε-PL biosynthesis without any adverse impact on growth in *S.* *albulus* among all the AdpA homologs studied in this paper, and the ε-PL yield of *S.* *albulus* SNA was approximately 3.6-fold that of the control strain. Only AdpA_*Sd*_ has been reported to have the regulatory roles in morphological differentiation and secondary metabolite toyocamycin biosynthesis in *S.* *diastatochromogenes* 1628 among the five AdpA proteins studied in this paper [68]. Therefore, our findings deepen insights into the regulatory roles of the *Streptomyces* AdpA family and reveal the functional similarity and discrepancy of AdpA homologs in *Streptomyces*, which may be connected with the similarity and discrepancy of AdpA homolog structures. On the one hand, the AdpA proteins studied in this paper possess sequence identities and structural similarities. The amino acid sequence of AdpA_*Sa*_ has identities with those of AdpA_*Sd*_ (99.05%), AdpA-SH (91.59%), AdpA_*Sn*_ (49.07%) and AdpA-C (88.83%). The amino acid sequences of the ThiJ/PfpI/DJ-1-like dimerization domain and AraC/XylS-type DNA-binding domain possess identities among the AdpA proteins studied in this paper, and these five AdpA proteins all retain the arginine residues at their positions corresponding to Arg-262 and Arg-266 that directly recognize the target DNA sequences in AdpA_*Sg*_ (Fig. 1) [67], which indicates that these five AdpA proteins may have functional similarities in AdpA dimerization and DNA binding. On the other hand, the UUA codons of *adpA* genes studied here differed in number/position (Fig. 1), and the expression of *adpA* is restricted to the translation level of the rare leucyl UUA codon [7]. Because the mature tRNA^Leu^_UAA_ of *Streptomyces* is present only after the activation of secondary metabolism and onset of morphological differentiation, the translation of TTA-containing genes can be interrupted by the absence of tRNA^Leu^_UAA_ during early stages of cell growth [79]. Considering that the transcription factors SlbR, ArfA, ArpA and BldD directly regulate the transcription levels of *adpA* genes in addition to the autoregulation of *adpA* in various *Streptomyces* species [66, 80–82], the synthesized strong promoter PermE* was used to express the *adpA* genes studied here in *S*. *albulus*. It’s recently reported that the posttranslational regulation is a possible new mechanism that may regulate AdpA protein abundance in *Streptomyces* cells [83]. Consequently, we surmise that regulation at the translational level by the UUA codon and posttranslational regulation might cause different AdpA protein abundances in the mutants studied in this paper. The specific mechanism by which the structures of AdpA homologs affect their functions needs to be further studied. Furthermore, the results of plate streaking (Additional file 1: Fig. S2E) showed that the introduction of plasmid pSET152 restricted morphological differentiation in *S.* *albulus*, which might result from the inactivation of the gene (SCF4126) encoding a putative pirin-like protein caused by the integration of pSET152 into the putative ϕC31 attB site within this gene. It was reported that some phenotypic changes and a significant drop in spiramycin production were found in *Streptomyces* *ambofaciens* ATCC 23,877 because the integration of the ϕC31-based integrative plasmid caused the inactivation of the gene SAM23877_RS18305 (*pirA*) encoding pirin [84].

To clarify the mechanism by which AdpA_*Sn*_ affects ε-PL biosynthesis in *S.* *albulus*, we studied the influences of AdpA_*Sn*_ on the key genes involved in ε-PL biosynthesis and the degree of ε-PL polymerization*.* The results of RT–qPCR and MST indicate that AdpA_*Sn*_ activates the transcription of *pyk2* and *pepc* but represses the transcription of *zwf* by directly binding to their promoter regions with similar dissociation constants. ε-PL biosynthesis is involved in EMP, PPP, the anaplerotic metabolic pathway, the TCA cycle, DAP and ε-PL assembly in *Streptomyces* (Fig. 6A) [51, 69, 70]. First, as the carbon flux flowing to the PPP is determined by G6PDH [71], the fact that AdpA_*Sn*_ directly represses the transcription of *zwf* may cause the carbon skeletons derived from glucose to be used more for glycolysis than for cell growth. It was reported that the deletion of either the *zwf1* or *zwf2* gene improved the production of actinorhodin and undecylprodigiosin (Red) with no influence on mycelium growth in *Streptomyces* *lividans* [85]. The *Streptomyces* *rimosus* M4018 strain obtained a higher production of OTC by the disruption of *zwf1* or *zwf2* [72]. Therefore, controlling the carbon flux of the PPP by adjusting the transcription of genes encoding G6PDH may be a useful way to promote secondary metabolites in *Streptomyces*, which indicates that the influence of AdpA_*Sn*_ on the ratio of carbon flux between the PPP and EMP might be one of the reasons for the enhanced ε-PL biosynthesis. Second, the effect of AdpA_*Sn*_ on the transcription level of *pyk2* (no distinct difference was found in the transcription level of *pyk1* between *S.* *albulus* SNA and *S.* *albulus* NK660) may cause more provision of carbon skeletons for the TCA cycle and a higher level of ATP for the regulation of Pls catalytic function, which may contribute to increasing ε-PL production. It was confirmed that the *pyk2*::Tn5062 transposon insertion mutant showed reduced polyketide coelimycin and Red yields in *S.* *coelicolor*, which indicates that the perturbation of central metabolism by adjusting the expression of genes encoding PK affects specialized metabolite biosynthesis [86]. Moreover, ε-PL synthesis depended on ATP, and the catalytic function of Pls was allosterically regulated by the concentration of intracellular ATP [87, 88]. Third, AdpA_*Sn*_ may prompt the replenishment of more TCA cycle intermediates for L-lysine biosynthesis and cause the generation of more carbon skeletons and ATP for ε-PL biosynthesis by directly activating the transcription of *pepc*. Finally, combined with the fact that the transcription levels of *ask*, *pls* and *hrdD* showed no significant differences between *S.* *albulus* SNA and *S.* *albulus* NK660 (Fig. 6B), all these results above demonstrate that AdpA_*Sn*_ may cause the redistribution of metabolic flux in central metabolism pathways by directly regulating the transcription of *zwf,* *pyk2* and *pepc*, which subsequently makes for more carbon skeletons and ATP for ε-PL biosynthesis in *S.* *albulus*. Furthermore, the molecular weight of ε-PL strongly affects its biological functions [38, 55]. ε-PL with less than 9 polymerization degrees has no obvious antibacterial activity, while high l-lysine polymerization degrees of ε-PL lead to an unpleasant bitter taste [55, 89]. Considering that the antibacterial activity and taste of ε-PL are consistent with its molecular weight, we studied the influence of AdpA_*Sn*_ on the ε-PL polymerization degree in *S.* *albulus*, and the results of MALDI-TOF–MS showed that *S.* *albulus* SNA and *S.* *albulus* NK660 produced ε-PL with the same polymerization degree of 19–33 l-lysine monomers (Fig. 9). This means that AdpA_*Sn*_ does not affect the polymerization degree of ε-PL in *S.* *albulus*, which agrees with the conclusion that Pls directly affects the molecular weight of ε-PL [76].

**Fig. 10:**
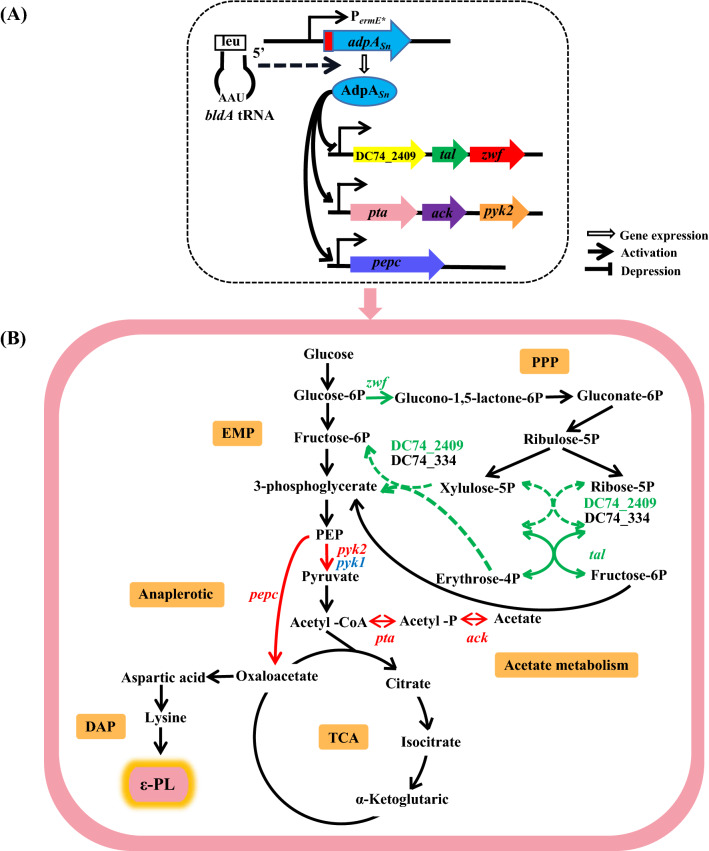
The proposed model showing the regulation of AdpA_*Sn*_ in central metabolism and acetate metabolism pathways. **A** Proposed model of the AdpA_*Sn*_-mediated regulatory network in *S.* *albulus* SNA. Solid lines indicate the direct regulation confirmed experimentally in this paper. Dashed lines indicate that *bldA* could affect the translation level of the UUA codon-containing gene *adpA*_*Sn*_. **B** The effects of AdpA_*Sn*_ on metabolic network and ε-PL biosynthesis are displayed. The seven target genes of AdpA_*Sn*_ are indicated in the simplified metabolic network, and the green, red and blue fonts or arrows stand for negative regulation, positive regulation and no regulation of AdpA_*Sn*_, respectively. The green dotted arrows mean these metabolic pathways may be negatively regulated because the transcription level change of the gene (DC74_334) encoding the isozyme of DC74_2409 is unknown

Moreover, we identified seven target genes in which AdpA_*Sn*_ directly regulated their expression in the primary metabolism pathways (Fig. 10). Because the upstream genes encoding phosphate acetyltransferase (*pta*) and acetate kinase (*ack*) are cotranscribed with *pyk2,* AdpA_*Sn*_ directly activates the transcription of *pta* and *ack* as well. The enzymes phosphate acetyltransferase and acetate kinase form the key pathway that is responsible for the interconversion between ADP, acetyl-CoA, orthophosphate and ATP, acetate, CoA in many bacteria [90, 91]. It was reported that Ack-Pta activity might be able to control the cellular concentration of acetyl-CoA, which plays a central role in carbon metabolism [90, 92, 93], and acetyl-phosphate, which acts as a global signal regulating the functions of proteins involved in biofilm development, flagella biosynthesis and assembly, type-I pilus assembly and colonic acid biosynthesis [94, 95]. Because the upstream genes encoding transketolase (DC74_2409) and transaldolase (*tal*) are cotranscribed with *zwf,* AdpA_*Sn*_ directly represses the transcription of the upstream gene encoding transketolase and *tal*. The nonoxidative branch of the pentose phosphate pathway in carbohydrate metabolism can provide precursors for the synthesis of aromatic amino acids, nucleic acids and fatty acids [96]. As important enzymes in the nonoxidative branch of the PPP, transketolase and transaldolase catalyze the two- and three-carbon fragment transfer from the ketose donor to the aldose acceptor and create a reversible link between the PPP and glycolysis [96, 97]. Consequently, quite interestingly, we identified seven target genes of AdpA_*Sn*_, which indicates that the direct regulation of AdpA_*Sn*_ involves both the oxidative part and nonoxidative branch of the pentose phosphate pathway, glycolytic pathway, anaplerotic metabolic pathway and Ack-Pta pathway of acetate metabolism; thus, AdpA_*Sn*_ plays an important role in the central metabolism and acetate metabolism pathways. Because of the importance of AdpA_*Sn*_ in the regulation of central metabolism and acetate metabolism pathways and the fact that AdpA_*Sn*_ promotes ε-PL biosynthesis mainly by regulating the transcription of the target genes in primary metabolism pathways but not the key genes (*ask* and *pls*) in the diaminopimelic acid pathway and ε-PL assembly, we hope this study will provide a reference for enhancing the production of ε-PL and other valuable secondary metabolites in *Streptomyces*.

## Conclusion

In summary, we characterized the positive regulatory effects in ε-PL biosynthesis and diversified regulation of the morphological differentiation of *Streptomyces* AdpA homologs and effectively promoted ε-PL biosynthesis and morphological differentiation in *S.* *albulus* by selecting AdpA_*Sn*_ from five AdpA homologs to be heterologously expressed. In addition, we clarified the mechanism by which AdpA_*Sn*_ affected ε-PL biosynthesis and its effect on the ε-PL polymerization degree in *S.* *albulus* and indicated that AdpA_*Sn*_ promotes ε-PL biosynthesis mainly by regulating the transcription of the target genes (*zwf*, *pyk2* and *pepc*) in primary metabolism pathways. Thereafter, we revealed the importance of AdpA_*Sn*_ in the regulation of central metabolism and acetate metabolism pathways by the identification of its seven target genes, which provides a reference that AdpA_*Sn*_ may possess the ability to promote the production of other valuable secondary metabolites in addition to ε-PL. These findings provide valuable insights into the regulatory roles of the *Streptomyces* AdpA family on ε-PL biosynthesis and morphological differentiation and supply an efficient strategy to promote the production of ε-PL and other valuable secondary metabolites in *Streptomyces*.

## Supplementary Information

**Additional file 1: Figure S1.** The upstream and downstream genes of different adpA genes and phylogenetic analysis of AdpA homologs. (A) Different adpA genes and their upstream and downstream genes in three *Streptomyces* species. The sequence identities between the encoded proteins of homologous genes with same color are indicated. (B) Phylogenetic analysis of the novel AdpASa, four heterologous AdpA homologs AdpASd, AdpA-SH, AdpASn, AdpA-C with NCBI BLASTP hits and well-studied ones. AdpASa is marked with the red hollow circle, while the four heterologous AdpA homologs AdpASd, AdpA-SH, AdpASn, AdpA-C are all marked with the blue hollow diamond. Different clades or subclades are indicated with different colors, and some bootstrap values of the phylogenetic tree are also displayed. **Figure S2.** Construction of *S.* *albulus* NKA and effect of AdpASa on specific ɛ-PL formation rate. (A) Schematic method for overexpressing adpASa in *S.* *albulus*. (B) Confirmation of the integration of adpASa gene into the genome of *S.* *albulus* by PCR. Lane M, DNA marker III; Lanes1-6, amplification products using primers SET-F/SET-R with gDNA from *S.* *albulus* SET, plasmid pSET152 DNA, gDNA from *S.* *albulus* NKA, plasmid pSET152-adpASa DNA, gDNA from *S.* *albulus* NK660 and ddH2O as templates, respectively. (C) Transcription levels of adpASa gene in *S.* *albulus* NK660 and *S.* *albulus* NKA by RT-qPCR analysis. ***P < 0.001 (Student's t-test). Error bars stand for the SD for three biological replicates. (D) Specific formation rates of ɛ-PL in *S.* *albulus* NKA and control strains (*S.* *albulus* SET and *S.* *albulus* NK660) cultured in fermentation medium for 100 h. Error bars stand for the SD for three biological replicates. (E) Phenotypes of *S.* *albulus* NK660 and *S.* *albulus* SET grown on MSF agar plates for 4 days. **Figure S3.** Construction of *S.* *albulus* SDA, *S.* *albulus* SHA, *S.* *albulus* SNA and *S.* *albulus* SCA. (A) Schematic method for expressing heterologous adpA genes in *S.* *albulus*. The blue arrow stand for heterologous adpA genes (adpASd, adpA-SH, adpASn, adpA-C). (B) Confirmation of the construction of mutants expressing heterologous adpA genes by PCR. Lane M, DNA marker III; Lanes1, 3, 5, 7, 9, 11, amplification products using gDNA from *S.* *albulus* SET, *S.* *albulus* SDA, *S.* *albulus* SHA, *S.* *albulus* SNA, *S.* *albulus* SCA and *S.* *albulus* NK660 as templates, respectively; Lanes 2, 4, 6, 8, 10, 12, amplification products using plasmid pSET152, pSET152-adpASd, pSET152-adpA-SH, pSET152-adpASn, pSET152-adpA-C and ddH2O as templates, respectively. And all the amplification products use the primers SET-F/SET-R. (C) RT-PCR results among *S.* *albulus* NK660, *S.* *albulus* SET and the four heterologous adpA genes expression mutants. Lane M, DNA marker III; Lanes D1-D10, amplification products using primers DadpA-F/DadpA-R with RNA from *S.* *albulus* NK660, cDNA from *S.* *albulus* NK660, RNA from *S.* *albulus* SET, cDNA from *S.* *albulus* SET, RNA from *S.* *albulus* SDA, cDNA from *S.* *albulus* SDA, gDNA from *S.* *albulus* SDA and ddH2O as templates, respectively. Lanes H1-H10, Lanes N1-N10 and Lanes C1-C10 are the amplification products respectively using primers HadpA-F2/HadpA-R, NadpA-BF/NadpA-BCR and CadpA-F/CadpA-R with the same templates and template orders as Lanes D1-D10. **Table S1.** Primers used in this study. **Table S2.** Accession number and length of the whole candidates used for phylogenetic analysis.

## Data Availability

All data generated or analyzed during the current study are included in this published article and its Additional file.
