# Supplementary material for: AdpA, a developmental regulator, promotes ε-poly-l-lysine biosynthesis in Streptomycesalbulus
Source: Microb Cell Fact. 2022 Apr 9;21:60. doi: 10.1186/s12934-022-01785-6 (PMC8994273; doi:10.1186/s12934-022-01785-6)
Supplement: Supplementary file 1 — Additional file 1: Figure S1. The upstream and downstream genes of different adpA genes and phylogenetic analysis of AdpA homologs. (A) Different adpA genes and their upstream and downstream genes in three Streptomyces species. The sequence identities between the encoded proteins of homologous genes with same color are indicated. (B) Phylogenetic analysis of the novel AdpASa, four heterologous AdpA homologs AdpASd, AdpA-SH, AdpASn, AdpA-C with NCBI BLASTP hits and well-studied ones. AdpASa is marked with the red hollow circle, while the four heterologous AdpA homologs AdpASd, AdpA-SH, AdpASn, AdpA-C are all marked with the blue hollow diamond. Different clades or subclades are indicated with different colors, and some bootstrap values of the phylogenetic tree are also displayed. Figure S2. Construction of S. albulus NKA and effect of AdpASa on specific ɛ-PL formation rate. (A) Schematic method for overexpressing adpASa in S. albulus. (B) Confirmation of the integration of adpASa gene into the genome of S. albulus by PCR. Lane M, DNA marker III; Lanes1-6, amplification products using primers SET-F/SET-R with gDNA from S. albulus SET, plasmid pSET152 DNA, gDNA from S. albulus NKA, plasmid pSET152-adpASa DNA, gDNA from S. albulus NK660 and ddH2O as templates, respectively. (C) Transcription levels of adpASa gene in S. albulus NK660 and S. albulus NKA by RT-qPCR analysis. ***P < 0.001 (Student's t-test). Error bars stand for the SD for three biological replicates. (D) Specific formation rates of ɛ-PL in S. albulus NKA and control strains (S. albulus SET and S. albulus NK660) cultured in fermentation medium for 100 h. Error bars stand for the SD for three biological replicates. (E) Phenotypes of S. albulus NK660 and S. albulus SET grown on MSF agar plates for 4 days. Figure S3. Construction of S. albulus SDA, S. albulus SHA, S. albulus SNA and S. albulus SCA. (A) Schematic method for expressing heterologous adpA genes in S. albulus. The blue arrow stand for heterologous [file 12934_2022_1785_MOESM1_ESM.pdf]

AdpA, a developmental regulator, promotes ε-poly-L-lysine biosynthesis in *Streptomyces albulus*

Rui Huang,[1] Honglu Liu,[1] Wanwan Zhao,[1] Siqi Wang,[1] Shufang Wang,[2]

Jun Cai,[1*] Chao Yang[1*]

[1]Key Laboratory of Molecular Microbiology and Technology, Ministry of Education, College of Life Sciences, Nankai University, Tianjin 300071, China

[2]Key Laboratory of Bioactive Materials, Ministry of Education, College of Life Sciences, Nankai University, Tianjin 300071, China

Rui Huang (E-mail: a993014540@163.com)

Honglu Liu (E-mail: 1120190490@mail.nankai.edu.cn)

Wanwan Zhao (E-mail: zw20070101@163.com)

Siqi Wang (E-mail: 17853153385@163.com)

Shufang Wang (E-mail: wangshufang@nankai.edu.cn)

[*]Correspondence to:

Jun Cai (Tel./ Fax: +86-22-23505964; E-mail: caijun@nankai.edu.cn)

Chao Yang (Tel./Fax: +86-22-23503866; E-mail: yangc20119@nankai.edu.cn)

Additional file 1

Fig. S1. The upstream and downstream genes of different *adpA* genes and phylogenetic analysis of AdpA homologs. (A) Different *adpA* genes and their upstream and downstream genes in three *Streptomyces* species. The sequence identities between the encoded proteins of homologous genes with same color are indicated. (B) Phylogenetic analysis of the novel AdpA$_{Sa}$, four heterologous AdpA homologs AdpA$_{Sd}$, AdpA-SH, AdpA$_{Sn}$, AdpA-C with NCBI BLASTP hits and well-studied ones. AdpA$_{Sa}$ is marked with the red hollow circle, while the four heterologous AdpA homologs AdpA$_{Sd}$, AdpA-SH, AdpA$_{Sn}$, AdpA-C are all marked with the blue hollow diamond. Different clades or subclades are indicated with different colors, and some bootstrap values of the phylogenetic tree are also displayed.

(A)

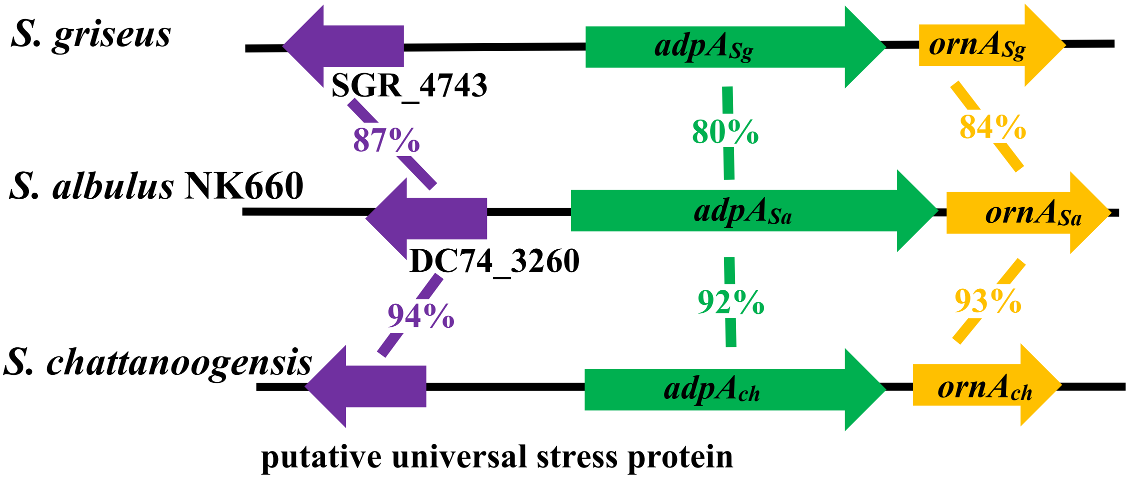

(B)

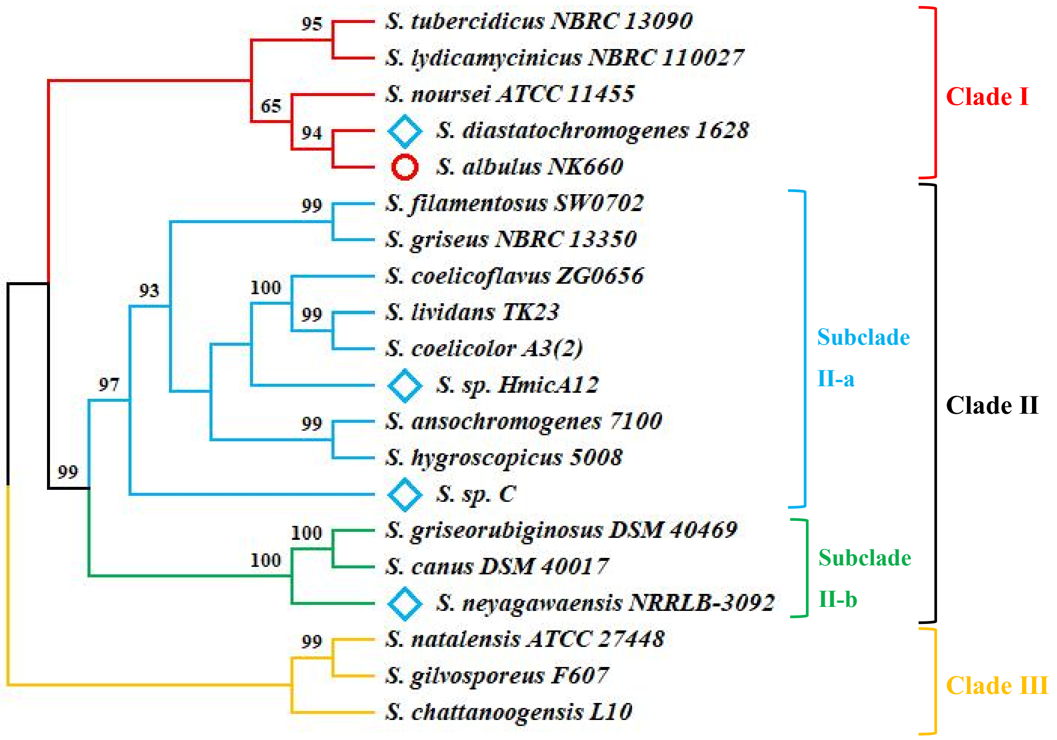

95
S. tubercidicus NBRC 13090
S. lydicamycinicus NBRC 110027
65
S. noursei ATCC 11455
94
S. diastatochromogenes 1628
S. albulus NK660
99
S. filamentosus SW0702
S. griseus NBRC 13350
93
100
S. coelicoflavus ZG0656
99
S. lividans TK23
S. coelicolor A3(2)
S. sp. HmicA12
97
99
S. ansochromogenes 7100
S. hygroscopicus 5008
99
S. sp. C
100
S. griseorubiginosus DSM 40469
100
S. canus DSM 40017
S. neyagawaensis NRRLB-3092
99
S. natalensis ATCC 27448
S. gilvosporeus F607
S. chattanoogensis L10
Clade I
Subclade II-a
Clade II
Subclade II-b
Clade III

Fig. S2. Construction of *S. albulus* NKA and effect of AdpA$_{Sa}$ on specific ε-PL formation rate. (A) Schematic method for overexpressing *adpA*$_{Sa}$ in *S. albulus*. (B) Confirmation of the integration of *adpA*$_{Sa}$ gene into the genome of *S. albulus* by PCR. Lane M, DNA marker III; Lanes1-6, amplification products using primers SET-F/SET-R with gDNA from *S. albulus* SET, plasmid pSET152 DNA, gDNA from *S. albulus* NKA, plasmid pSET152-*adpA*$_{Sa}$ DNA, gDNA from *S. albulus* NK660 and ddH_2O as templates, respectively. (C) Transcription levels of *adpA*$_{Sa}$ gene in *S. albulus* NK660 and *S. albulus* NKA by RT-qPCR analysis. ***P < 0.001 (Student's *t*-test). Error bars stand for the SD for three biological replicates. (D) Specific formation rates of ε-PL in *S. albulus* NKA and control strains (*S. albulus* SET and *S. albulus* NK660) cultured in fermentation medium for 100 h. Error bars stand for the SD for three biological replicates. (E) Phenotypes of *S. albulus* NK660 and *S. albulus* SET grown on MSF agar plates for 4 days.

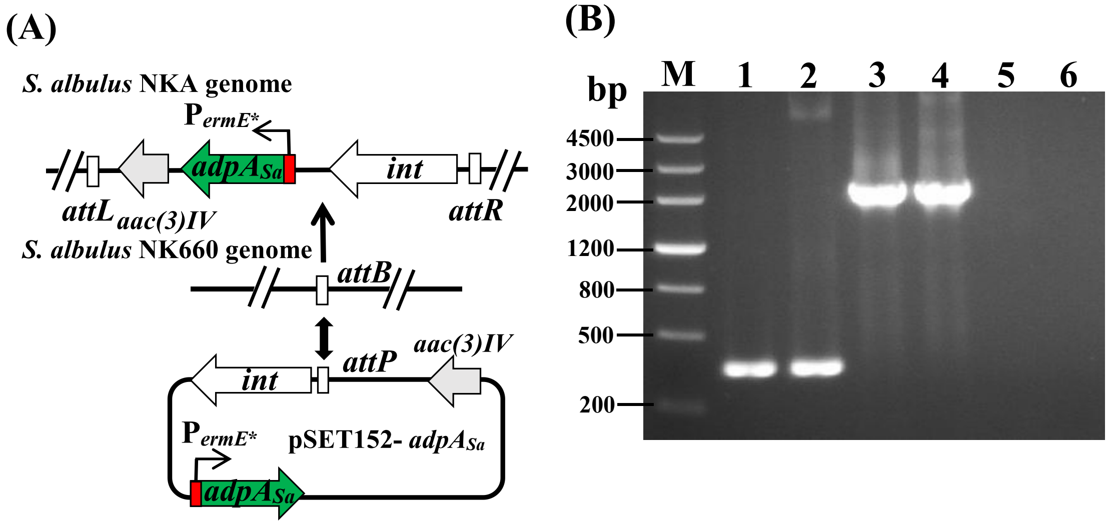

(C)

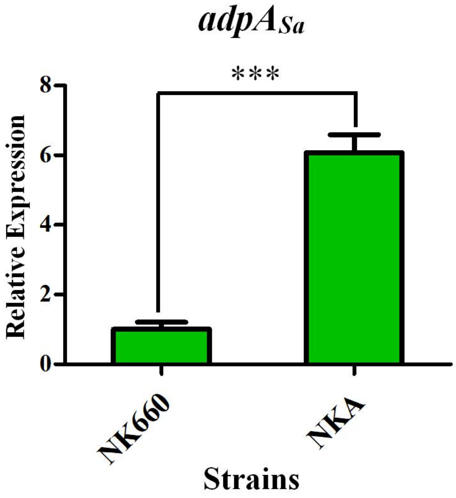

adpA Sa
***
8
6
4
2
0
Relative Expression
NK660
NKA
Strains

(D)

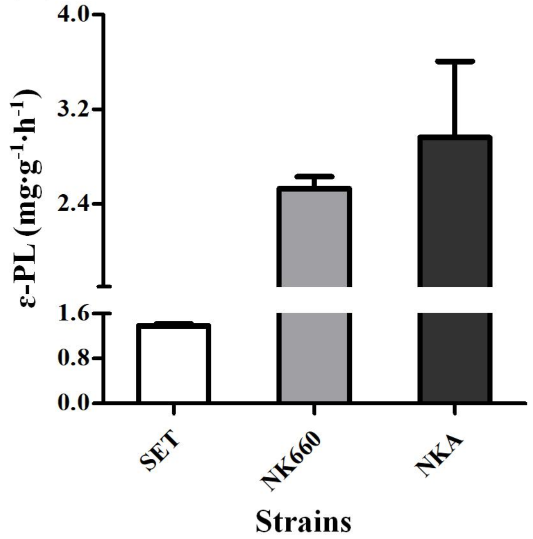

4.0
3.2
2.4
1.6
0.8
0.0
ε-PL (mg·g-1·h-1)
SET
NK660
NKA
Strains

(E)

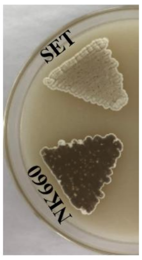

SET
NK660

Fig. S3. Construction of *S. albulus* SDA, *S. albulus* SHA, *S. albulus* SNA and *S. albulus* SCA. (A) Schematic method for expressing heterologous *adpA* genes in *S. albulus*. The blue arrow stand for heterologous *adpA* genes ($adpA_{Sd}$, *adpA-SH*, $adpA_{Sn}$, *adpA-C*). (B) Confirmation of the construction of mutants expressing heterologous *adpA* genes by PCR. Lane M, DNA marker III; Lanes1, 3, 5, 7, 9, 11, amplification products using gDNA from *S. albulus* SET, *S. albulus* SDA, *S. albulus* SHA, *S. albulus* SNA, *S. albulus* SCA and *S. albulus* NK660 as templates, respectively; Lanes 2, 4, 6, 8, 10, 12, amplification products using plasmid pSET152, pSET152-$adpA_{Sd}$, pSET152-*adpA-SH*, pSET152-$adpA_{Sn}$, pSET152-*adpA-C* and ddH_2O as templates, respectively. And all the amplification products use the primers SET-F/SET-R. (C) RT-PCR results among *S. albulus* NK660, *S. albulus* SET and the four heterologous *adpA* genes expression mutants. Lane M, DNA marker III; Lanes D1-D10, amplification products using primers DadpA-F/DadpA-R with RNA from *S. albulus* NK660, cDNA from *S. albulus* NK660, RNA from *S. albulus* SET, cDNA from *S. albulus* SET, RNA from *S. albulus* SDA, cDNA from *S. albulus* SDA, gDNA from *S. albulus* SDA and ddH_2O as templates, respectively. Lanes H1-H10, Lanes N1-N10 and Lanes C1-C10 are the amplification products respectively using primers HadpA-F2/HadpA-R, NadpA-BF/NadpA-BCR and CadpA-F/CadpA-R with the same templates and template orders as Lanes D1-D10.

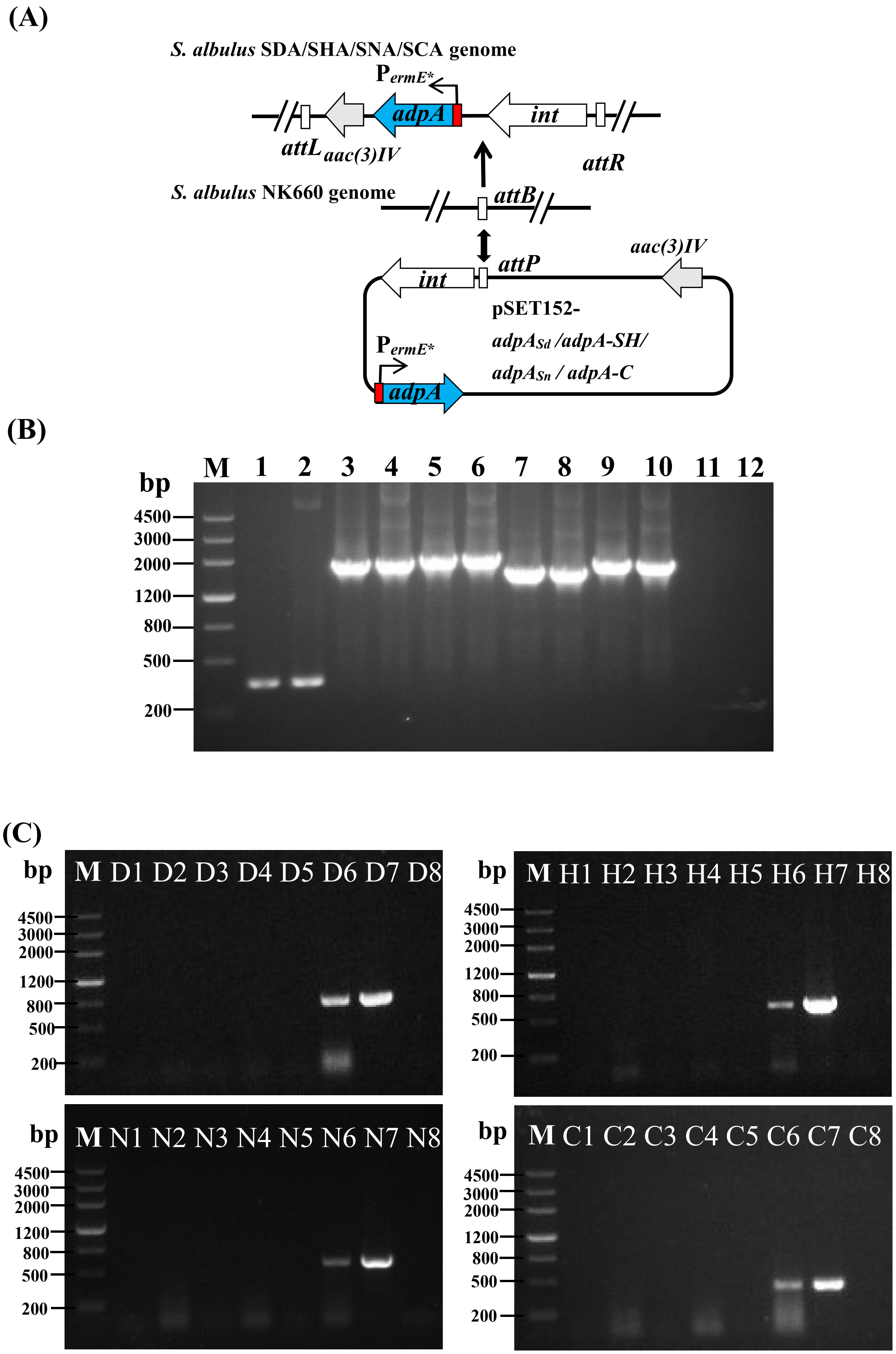
(A)
S. albulus SDA/SHA/SNA/SCA genome
PermE*
adpA
int
attL
aac(3)IV
attR
S. albulus NK660 genome
attB
attP
aac(3)IV
int
pSET152-
adpASd /adpA-SH/
adpASn / adpA-C
PermE*
adpA
(B)
bp
M 1 2 3 4 5 6 7 8 9 10 11 12
4500
3000
2000
1200
800
500
200
(C)
bp
M D1 D2 D3 D4 D5 D6 D7 D8
4500
3000
2000
1200
800
500
200
bp
M H1 H2 H3 H4 H5 H6 H7 H8
4500
3000
2000
1200
800
500
200
bp
M N1 N2 N3 N4 N5 N6 N7 N8
4500
3000
2000
1200
800
500
200
bp
M C1 C2 C3 C4 C5 C6 C7 C8
4500
3000
2000
1200
800
500
200

Table S1: Primers used in this study.

Primers	Sequence (5’-3’)
**Construction of *S. albulus* NK660 and *E. coli* BL21(DE3) derivatives**	
SET-F	GTGCTGCAAGGCGATTAAGTTGG
SET-R	AGCTGGCACGACAGGTTTCC
PermE*-XF	GGGCTGCAGGTCGACTCTAGAGACGTCCATGCGAGT<br>GTCCG
PermE*-adpAR	TCGCAGTAGGGCACAACCAT<br>TGGGGTCCTCCTGTGGAGTG
adpA-F	CACTCCACAGGAGGACCCCAATGGTTGTGCCCTACTG<br>CGA
adpA-R	CGCGGCCGCGGATCC CATATG<br>TCACCCTACGGGGCGCTCCC
PermE*+DadpA-XR	CGCGGCCGCGGATCCCATATGCTACGGGGCGCTGCG<br>CTGAC
PermE*+HadpA-XR	CGCGGCCGCGGATCCCATATG<br>CTACGGAGCCGTCCGCGGTC
PermE*+NadpA-XR	CGCGGCCGCGGATCCCATATG<br>TCAGTGCGTGGTCGTGTCGG
PermE*+CadpA-XR	CGCGGCCGCGGATCCCATATG<br>CTACGCGTGCTCGCGCAGCA
NadpA-OF	GTGCCGCGCGGCAGCCATATGCTGAAGAACGTAGCC<br>GCGGT
NadpA-OR	TTGTCGACGGAGCTCGAATTCTCAGTGCGTGGTCGTG<br>TCGG
**MST**	
pyk2-F	CGTGCTGTGTCTCCTGTGCT
pyk2-R	GCGTGGTCTCCGTCCATCCG
pepc-F	GTGGTGTCCCTCGTCGGGGG
[1, 2]*pepc*-R	AGGTGCGGCTCCTTGCAAGG
zwf-F	GCGGAGATAAGGCCAGCAAG
zwf-R	CGCTCAGTCGGTGATTTCAG
**RT-qPCR, RT-PCR**	
DadpA-F	GAGGACCCCAATGAGCCA
DadpA-R	GTAACGACCTGTCCAGGTAG
HadpA-F2	GCTATCCGTCGGTCCATGTG
HadpA-R	ACGGCTTCTCCGAGAGCTTC
NadpA-BF	ACGTCCATCCATTCGAACTC
NadpA-BCR	CCGGTCGTTCGATGTACTGG
CadpA-F	GAGTTCCACGCTACCGACTG
CadpA-R	TGCAGGCACAGGTCGATTCC
HrdB-F	CGAGTCCGAGTCTGTGATGGCG

HrdB-R	CGAGGATTTGGTTGAGGCTGCG
NKadpA-qF	CTACCTGGACAGGTCGTTAC
NKadpA-qR	ACCTCGTCCACCGAATAGTC
HrdD-F	TCTACGCCCAGCAGATCCT
HrdD-R	GGTTGGACCGGATGAAGAC
Pls-F	GCCGCCTACGGACTGACCC
pls-R	CACGGCGGCGGTGACGAGGAGGGA
Ask-8F	AAGAAGAACGGCAACCAG
Ask-7/8/9R	TGAAGGACTGCGCCTCATGG
Pepc-F	GCAACTTCCTCTCCAACGTC
Pepc-R	CTCGTGTTCGGCCTTGAT
PYK2-F	GCCGTTCCAAAATCGTCTG
PYK2-R	GCCTCGATCAGCGTCTTCA
PYK1-F	GGGATTCTTCGGATGCTTTG
PYK1-R	AAGATCGTCTGCACACTAGG
G6PDH-4F	GAGCTCAACCGGATCGTC
G6PDH-4R	GCTCGAACATGGTGTTGG
p9	GTGGCCCGCTTCAACATGAG
p10	GTGCCGCAGATGTGCTTGTC
p7	GCGACAACGACATGCGAGAG
p6	CAAGGGCGTCAGCTACGAAC
p8	TCCCGTGGCTCATGTTGAAG
P1	TACGTGTGGACGCACGACTC
p2	TTCGTGTCCCGTGTGGACAG
p3	CGGCTCGAACATGGTGTTGG
p4	TCGATGGACTTCGCGTACGG
p5	TCGTGCGAGCATTTCGTCTG

Table S2: Accession number and length of the whole candidates used for phylogenetic analysis.

Organism	Accession No.	Length (aa)
S. griseus NBRC 13350	BAA86265.1	405
S. coelicolor A3(2)	CAB87229.1	398
S. diastatochromogenes 1628	AFX97763.1	420
S. sp. HmicA12	WP_018531726.1	441
S. neyagawaensis NRRLB-3092	WP_055538474.1	324
S. sp.C	WP_007264197.1	390
S. albulus NK660	AIA03759.1	501
S. lividans TK23	ACJ04048.1	398
S. hygroscopicus 5008	AEY89567.1	405
S. chattanoogensis L10	ACY78399.1	429
S. ansochromogenes 7100	ABY86620.1	407
S. canus DSM 40017	KUN72461.1	277
S. griseorubiginosus DSM 40469	KUN60558.1	282
S. filamentosus SW0702	AIR95874.1	405
S. coelicoflavus ZG0656	EHN73017.1	396
S. noursei ATCC 11455	ANZ18541.1	501
S. lydicamycinicus NBRC 110027	GAO09070.1	426
S. tubercidicus NBRC 13090	GFE39693.1	441
S. natalensis ATCC 27448	KIZ18815.1	429
S. gilvosporeus F607	ARF55436.1	429
